# Effects of Emotional Stimulations on the Online Operation of a P300-Based Brain–Computer Interface

**DOI:** 10.3389/fnhum.2021.612777

**Published:** 2021-02-26

**Authors:** Minju Kim, Jongsu Kim, Dojin Heo, Yunjoo Choi, Taejun Lee, Sung-Phil Kim

**Affiliations:** Department of Biomedical Engineering, Ulsan National Institute of Science and Technology, Ulsan, South Korea

**Keywords:** emotional stimulation, brain-computer interface, P300, ERP, auditory stimulus

## Abstract

Using P300-based brain–computer interfaces (BCIs) in daily life should take into account the user’s emotional state because various emotional conditions are likely to influence event-related potentials (ERPs) and consequently the performance of P300-based BCIs. This study aimed at investigating whether external emotional stimuli affect the performance of a P300-based BCI, particularly built for controlling home appliances. We presented a set of emotional auditory stimuli to subjects, which had been selected for each subject based on individual valence scores evaluated *a priori*, while they were controlling an electric light device using a P300-based BCI. There were four conditions regarding the auditory stimuli, including high valence, low valence, noise, and no sound. As a result, subjects controlled the electric light device using the BCI in real time with a mean accuracy of 88.14%. The overall accuracy and P300 features over most EEG channels did not show a significant difference between the four auditory conditions (*p* > 0.05). When we measured emotional states using frontal alpha asymmetry (FAA) and compared FAA across the auditory conditions, we also found no significant difference (*p* > 0.05). Our results suggest that there is no clear evidence to support a hypothesis that external emotional stimuli influence the P300-based BCI performance or the P300 features while people are controlling devices using the BCI in real time. This study may provide useful information for those who are concerned with the implementation of a P300-based BCI in practice.

## Introduction

A brain–computer interface (BCI) provides a direct communication channel between people and external environments without any involvement of muscles by translating brain signals directly into the commands (Wolpaw et al., 2000, 2002). Due to this capacity, BCIs can provide an alternative means of communication with the external world for those who are suffering from severe neurological disorders, such as amyotrophic lateral sclerosis, spinal cord injury, or brainstem stroke (Birbaumer and Cohen, 2007). Not only as a means for communication with the external world, BCIs can also be used to restore, enhance, supplement, and improve lost central nervous system (CNS) functions as well as to provide a decent research tool (Brunner et al., 2015). In particular, non-invasive BCIs based on electroencephalography (EEG) have been widely used due to their high temporal resolution and relatively low cost (Nicolas-Alonso and Gomez-Gil, 2012).

Brain-computer interfaces can be classified into several categories such as active, reactive, and passive BCIs (Zander and Kothe, 2011). Active BCIs elicit brain signals such as sensorimotor rhythms by self-paced and voluntary mental activity. Reactive BCIs induce brain signals such as event-related potentials (ERPs) or steady-state visually evoked potentials (SSVEPs) by providing external stimuli in a synchronous manner. Passive BCIs detect brain signals to infer various mental states. Among reactive BCIs relying on ERPs, P300-based BCIs have been the most widely investigated, where P300 refers to one of the ERP components induced by the oddball task paradigm (Sara et al., 1994). For instance, a P300-based BCI implements an oddball task with the visual arrangement of letters in a matrix form and enables one to select and type a letter using brain activity only (Farwell and Donchin, 1988). It has been further expanded for device control by selecting a target function amid available control functions using brain activity (Aloise et al., 2010; Carabalona et al., 2010; Corralejo et al., 2014; Halder et al., 2015; Miralles et al., 2015; Schettini et al., 2015; Pinegger et al., 2016; Zhang et al., 2017). This type of BCI, potentially combined with the Internet of things (IoT), is especially useful for those with severe neurological disorders to operate living goods such as home appliances (Aydin et al., 2016; Zhong et al., 2019).

To bring BCIs to one’s daily life for efficient communications and control of devices (Wolpaw et al., 2000), however, a number of issues need to be resolved. One of them is the fact that the BCI users are likely to be exposed to virtually all kinds of stimulations from environments, which can contribute unexpected and undefined sources of noise to EEG. In particular, the BCI users would undergo dynamically changing states of emotions driven by external and internal events, which would increase a chance to temporarily distort or alternate EEG patterns, affecting the performance of BCIs. This is particularly crucial for P300-based BCIs, because a number of ERP components (e.g., late positive potentials) are known to be related to emotional states and possibly overlapped with P300 (Schupp et al., 2000; Olofsson et al., 2008; Hajcak et al., 2010). For instance, Mehmood and Lee (2015) investigated ERPs during the perception of emotional visual stimuli (happy, scared, calm, and sad) and observed the occurrence of P300 at occipital and parietal regions. Also, Conroy and Polich (2007) reported that the frontal P300 amplitude varied with valence using emotional stimuli provided in an oddball paradigm.

Recently, a number of studies investigated the effect of using emotional stimuli as targets for P300-based BCIs. Zhao et al. (2013) demonstrated that P300-based BCIs using emotional faces as target stimuli showed higher performance than using non-face objects or neutral faces, due to the addition of ERP components of human face encoding and emotion processing to those of target recognition, which enhanced the discrimination of ERPs for targets. Onishi and colleagues (Onishi et al., 2017; Onishi and Nakagawa, 2019) used emotional auditory stimuli in a certain range of valence for P300-based BCIs and suggested that auditory stimuli of positive valence improved BCI performance. In addition, Fernandez-Rodríguez et al. (2019) reported that using emotional or neutral pictures resulted in better performance than using letters as a BCI stimulus, which was supported by more preferable evaluations by the users on neutral and positive emotional pictures. Lu et al. (2019) developed an audiovisual P300 speller equipped with emotional visual and auditory stimuli, which resulted in an improvement of performance. All of these studies, however, used emotional stimuli as targets for the oddball paradigm, which users attended to all the time. However, when we take the scenario of daily use of BCIs into consideration, external emotional stimuli would be more likely irrelevant to BCI control of devices, which the BCI users need to ignore but can be affected—e.g., the sound of a laugh or a crash. In this context, little is known about the effect of external emotional stimuli on P300-based BCIs, not as target stimuli used as a part of BCIs, but as ambient stimuli irrelevant to BCIs.

Therefore, this study aims to investigate whether external emotional stimuli irrelevant to the oddball paradigm influence the performance of a P300-based BCI used for controlling home appliances. To modulate one’s emotional states, we used external emotional auditory stimuli concurrently with the oddball task in which visual device control icons were used as target or non-target stimuli. Thus, the BCI user selected a visual target while receiving auditory emotional stimuli irrelevant to visual stimuli. The emotional auditory stimuli used in this study were selected from the International Affective Digitized Sounds (IADS) (Bradley and Lang, 2007). To address individual differences in emotional responses to a given emotional auditory stimulus, we sorted a particular set of auditory stimuli for each user through a precedential behavioral experiment. To examine the effect of emotional changes on practical use of BCIs, we built an online P300-based BCI system that controlled an electric light device and examined the real-time effect of emotional stimuli on the users’ performances of controlling the electric light via the BCI system.

## Materials and Methods

### Participants

Seventeen healthy subjects participated in the study (7 Female, ages 22–28 with mean 24.61 ± 1.58). For a fair comparison of BCI outcomes, the age range in this study was selected similar to the previous BCI studies (Zhao et al., 2013; Lian et al., 2017; Onishi et al., 2017; Voznenko et al., 2018; Fernandez-Rodríguez et al., 2019). All subjects had normal or were corrected to normal vision and had no history of neurological or psychiatric disorders. All subjects gave informed consent for this study, approved by the Ulsan National Institutes of Science and Technology, Institutional Review Board (UNIST-IRB-18-08-A).

### Data Acquisition and Preprocessing

The scalp EEG data of subjects were acquired from 31 active wet electrodes (FP1, FPz, FP2, F7, F3, Fz, F4, F8, FC5, FC1, FC2, FC6, T7, C3, Cz, C4, CP5, T8, CP1, CP2, CP6, P7, P3, Pz, P4, P8, O1, Oz, and O2), using a standard EEG cap placed on the head following the 10–20 system of American Clinical Neurophysiology Society Guideline 2. Reference and ground electrodes were placed on mastoids of the left and right ears, respectively. The impedance of all electrodes was kept below 5 kΩ. EEG signals were amplified by a commercial EEG amplifier (anti-CHamp, Brain Product GmbH, Germany) and sampled at 500 Hz.

In our study, EEG signals were preprocessed as follows. First, a raw EEG signal was high-pass filtered above 0.5 Hz. Then, a bad EEG channel was detected and removed if more than 70% of all other channels showed a cross-correlation lower than 0.4 with that channel after being band-pass filtered through 0.5 to 1 Hz (Bigdely-Shamlo et al., 2015). This process removed four channels on average across subjects. Potential noise components from the reference were removed by using the common average reference (CAR) technique. The re-referenced EEG signal was low-pass filtered below 50 Hz. Then, artifacts were eliminated by the artifact subspace reconstruction (ASR) method (Mullen et al., 2015; Chang et al., 2018). Finally, the signal was low-pass filtered again below 12 Hz for the ERP analysis.

### Experimental Setup

The experiment was conducted twice on two different days in each subject, with an interval of 6–8 days between the experiments. In the first experiment, a pre-survey was taken for selecting emotion-induced sounds used as individual auditory stimuli for each subject. In the second experiment, subjects performed an online P300-based BCI session to control an electric light device while listening to the set of sounds selected in the first experiment. Afterward, they took a post-survey again for the emotion-induced sounds used in the BCI session.

#### Sound Samples Selection

We selected sound stimuli for individual subjects, used for inducing positive and negative emotions in them during the operation of the P300-based BCI; 100 emotional sound samples were prepared initially from the International Affective Digitized Sounds, the 2nd edition (IADS-2) (Bradley and Lang, 2007) based on the reported mean valence rating: 50 highest mean valence rating (Supplementary Table 1) and 50 lowest mean valence rating (Supplementary Table 2). These samples included natural sounds made by people, animals, and objects that are commonly experienced in daily life (Supplementary Table 1 and Supplementary Table 2). For each of the sound samples, the survey in the first experiment asked each subject to report how strongly they felt an emotion by scoring emotional response in each of the two emotional dimensions: valence and arousal. The score was scaled between −100 and 100 in each dimension. We informed subjects to score valence toward −100 if they felt strongly negative by the sound and toward +100 if they felt strongly positive. Also, we informed subjects to score arousal toward −100 if they were weakly aware of an emotion and toward +100 means if they were strongly aware of an emotion. The survey questions were provided to subjects in the text form.

**FIGURE 1:**
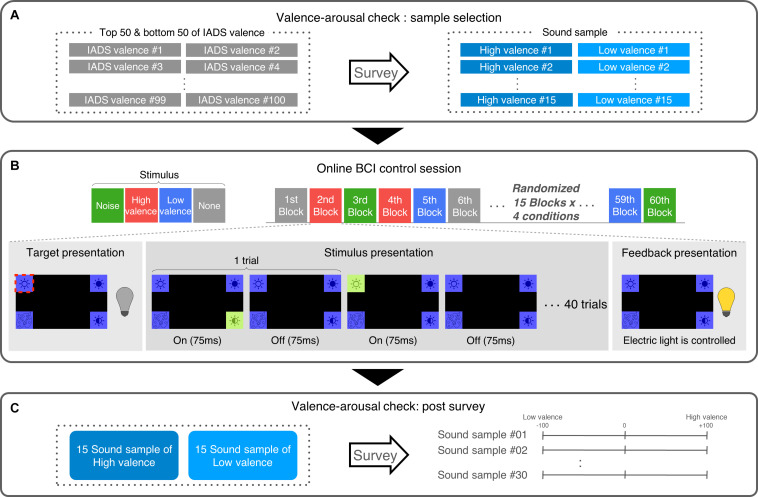
Experimental protocol. **(A)** Subjects reported the valence and arousal level of 100 IADS sound samples. From the reports, a total of 30 samples were selected for each subject (15 highest valence + 15 lowest valence values). **(B)** Approximately a week later, subjects revisited and conducted a P300 BCI control experiment while exposed to emotional auditory stimuli with four conditions (high valence, low valence, noise, and none). **(C)** After the P300 BCI experiment, subjects again rated the valence and arousal level of the sound samples that they heard during the BCI experiment.

In each subject, after the first experiment, we selected the 15 sound samples from each high valence (HV) and low valence (LV) group showing the largest absolute valence scores along with positive arousal scores (Figure 1A).

#### Online BCI Operation

In the second experiment, before the online P300-based BCI session, subjects performed a training session. The training session consisted of 40 blocks. Each block started with a fixation period for 500 ms where a fixation cross appeared at the center of the screen, followed by the random presentation of four visual stimuli located at each of the four corners of the screen. The stimuli were designed as a purple square with an icon indicating a control function of the electric light device. When highlighted, the color of square was changed to light green (Figure 1B). Subjects were given the information about which of the four stimuli was a target and asked to gaze at it through the block. Then, a trial began by highlighting one of the stimuli randomly for 75 ms followed by an inter-trial interval of 75 ms. There were 40 trials per block—four stimuli were highlighted exactly 10 times each in a random order, which led to 6 s of stimuli presentation. Including a fixation period, 4 s of target presentation and 4 s of feedback presentation and 1 s of inter-block interval, one block lasts 15.5 s resulting in 10.3 min of the training session. Note that no auditory stimulus was given to subjects during training.

After the training session was over, we epoched the acquired EEG data according to the stimulus information by distinguishing each stimulus as a target or non-target. Note that there was an overlap between successive epochs because the length of an epoch was set to −200 to 600 ms in this study which was longer than the length of a trial. This was originally designed for the development of online P300-based BCIs in our previous studies and shown to work properly (Kim et al., 2019). Then, we obtained ERPs in response to the target or the non-target in each block by taking average of EEG in the corresponding epoch over trials. From these ERPs, we extracted features from the P300 component as well as other potential components by taking out ERP amplitude values between 150 and 600 ms after stimulus onset. The features were then used to train a classifier based on support vector machine (SVM) with a linear kernel and penalty parameter C as 1, which discriminated between target and non-target. Note that there were 40 training samples in the target class and 120 samples in the non-target class, respectively. These data were imbalanced, possibly posing a problem for classification. Our previous study (Lee et al., 2020) showed that adjusting the penalty parameter C could resolve the problem of imbalance slightly, but the resulting improvement in accuracy was only marginal. According to this study, we did not adjust C in the online BCI experiment. In addition, during online BCI operation, one of the four stimuli that was closest to the target class based on SVM score was decided as a target.

With a P300-based BCI containing the trained classifier, subjects performed the online session to control an electric light device (Phillips hue 2.0, Phillips, Netherlands). The online session consisted of 60 blocks with four auditory conditions: HV sound presentation (HV), LV sound presentation (LV), noise sound presentation (Noise), and no sound (None). As a noise sound, we used a recording of ambient daily sounds mixed with human voices, dishes, and objects clattering. All sound samples were 6 s long so that it could be played in the same duration as the 6-s visual stimulation length. Subjects listened to the auditory stimulus through earphone at a sound level of 61 dB on average. There were 15 blocks in each of the four conditions. The order of the blocks was randomized. The composition of a block was same as that in training session, except for feedback presentation. In each block, subjects were given the information of which control command (out of four) they should operate and selected it using the BCI through 40 trials of the stimulus presentation in a block. The four commands included light on, light off, color change, and brightness change. After the block, subjects received feedback immediately from the real-time operation of the electric light device located in front of them according to the functional command generated by the BCI, regardless of the correctness of the operation (Figure 1B). Unlike automatic progress of the experiment in training session, the progress to the next block was done manually, one block lasted 20 s to 35 s, and the entire online session took approximately 20 min.

After the online session, subjects conducted a *post hoc* survey for the selected sound stimuli used in the session with the same scoring scheme as in the first experiment (i.e., −100 to 100 for valence and arousal each) (Figure 1C). This post survey was designed to examine how much emotional responses to the selected sound samples changed before and after the online BCI session.

### Data Analysis

#### ERP Analysis

We analyzed ERPs for the target stimuli obtained from the online test session across different auditory conditions. Specifically, we focused on the amplitude of a positive peak that was defined as the highest amplitude within a time window from 250 to 500 ms after stimulus onset. We also measured the latency of this peak in each ERP. To examine whether these ERP features were different across the four conditions, we applied repeated measures ANOVA (rmANOVA) for each ERP feature at each channel. Note that the number of subjects (i.e., samples) tested varied across channels due to individual differences of bad channel removal results (see Table 1). Also, the channel FT10 was completely excluded in this ERP analysis because this channel was removed in every subject except for one subject, which was due to problem of the corresponding electrode cap used in the experiment.

**TABLE 1:** The statistical test results of differences in the P300 peak amplitude and latency between emotional conditions (rmANOVA). The values that showed significance (*p* < 0.05) were highlighted in bold.

Channel	Amplitude		Latency		The number of subject
	*F*	*p*-value	*F*	*p*-value	
F3	**2.9188**	**0.0442**	0.9812	0.4101	16
Fz	1.1825	0.3263	0.7303	0.5390	17
F4	0.4108	0.7460	0.0348	0.9912	17
FC1	1.4411	0.2425	0.6397	0.5932	17
FC2	1.8931	0.14543	1.6373	0.1951	15
C3	2.1072	0.1116	2.4873	0.0717	17
Cz	0.1228	0.9462	0.3986	0.7546	17
C4	0.3269	0.8059	0.2995	0.8256	16
CP1	0.4269	0.7346	1.6363	0.1934	17
CP2	0.2813	0.8386	0.1964	0.8983	17
P3	0.2955	0.8285	1.0302	0.3876	17
Pz	0.6687	0.5754	0.0560	0.9823	17
P4	1.609	0.1996	1.0347	0.3857	17
O1	2.2360	0.0980	1.0720	0.3712	15
Oz	0.5893	0.6254	0.2911	0.8316	15
O2	1.1	0.3590	1.7083	0.1788	16

#### BCI Performance Analysis

Using the BCI control results from the online test session, we calculated accuracy given by the ratio of the number of blocks with correct target selection to the number of all blocks (i.e., 60). After obtaining accuracy of all subjects for each condition, we divided subjects into two groups according to the extent to which the presence of emotional stimuli affected subjects’ BCI control: a large difference (LD) and small difference (SD) groups. The LD group consisted of subjects who showed an increase or decrease of accuracy in either the HV or LV conditions by more than 10% compared to the None condition. The SD group consisted of the rest subjects. Since 15 blocks were conducted for each condition, one correct (or wrong) selection would cause the change of accuracy as much as 6.67%. Compared to None, more than one correct or wrong selection in either HV or LV was deemed to be a large difference in this study, as one or less correct or wrong selection in both HV and LV than in None would not sufficiently pronounce a difference of accuracy. Therefore, we set 10% of accuracy as a criterion to discriminate subject groups into the LD and SD groups. This division was intended to observe whether those who were influenced more by emotional stimuli regardless of the valence of emotion (HV or LV) showed different tendency compared to others. There were nine subjects in the LD group, and 8 in the SD group, respectively. Then, we compared BCI control accuracy as well as ERP features (see section “ERP Analysis”) and emotional EEG features (see section “Emotional EEG Analysis”) between the four conditions within each group. This further analysis was conducted to examine whether we could observe any influence of emotional stimuli on the BCI operation if we sharpened our focus on a certain group of individuals.

#### Emotional EEG Analysis

We analyzed EEG characteristics reflecting overt emotional responses to auditory stimuli during the operation of the BCI. Specifically, we examined frontal alpha asymmetry (FAA) that has been well known to represent valence (Coan and Allen, 2003). FAA was calculated by asymmetry between left and right hemisphere alpha-band power of EEG. In this study, FAA was determined as follows:

(1) FAA=10(ln(Power)right-ln(Power)left)

where Power_*left*_ was the average power of alpha band (8–14 Hz) at channel FP1, F3 and F7; and Power_*right*_ was the average power of the same frequency band at channel FP2, F4, and F8. We measured FAA from EEG data in each condition in each subject. Then, we compared FAA across the four conditions using rmANOVA.

## Results

### Survey Results

**FIGURE 2:**
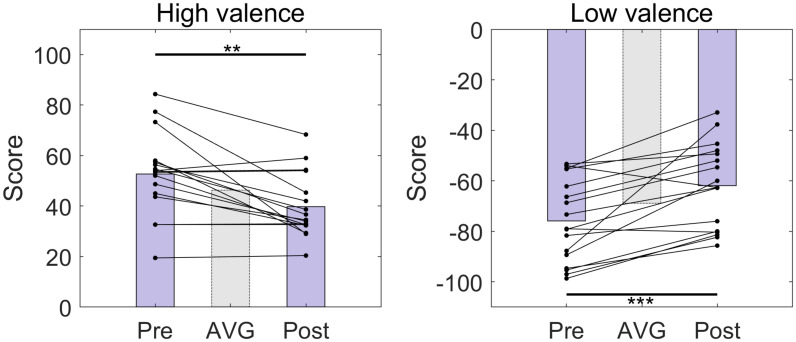
The distributions of the valence scores of high-, and low-valence stimuli used in the experiment before (Pre) and after (Post) the online BCI session. The bars indicate the average valence scores. AVG, average over all pre- and post-session scores. There were 15 high-valence and 15 low-valence stimuli, respectively. There was an approximately 1-week interval between pre- and post-session. **p < 0.01, ***p < 0.001, paired *t*-test.

We compared the valence scores from the survey of a set of 30 sound samples selected for each subject taken before and after the online BCI session (Figure 2). There was no instance that the sign of the valence scores was altered for any of the samples. However, the absolute values of the valence scores significantly decreased after the online BCI session (HV: *p* = 0.0012; LV: *p* < 0.001).

### ERP Differences

**FIGURE 3:**
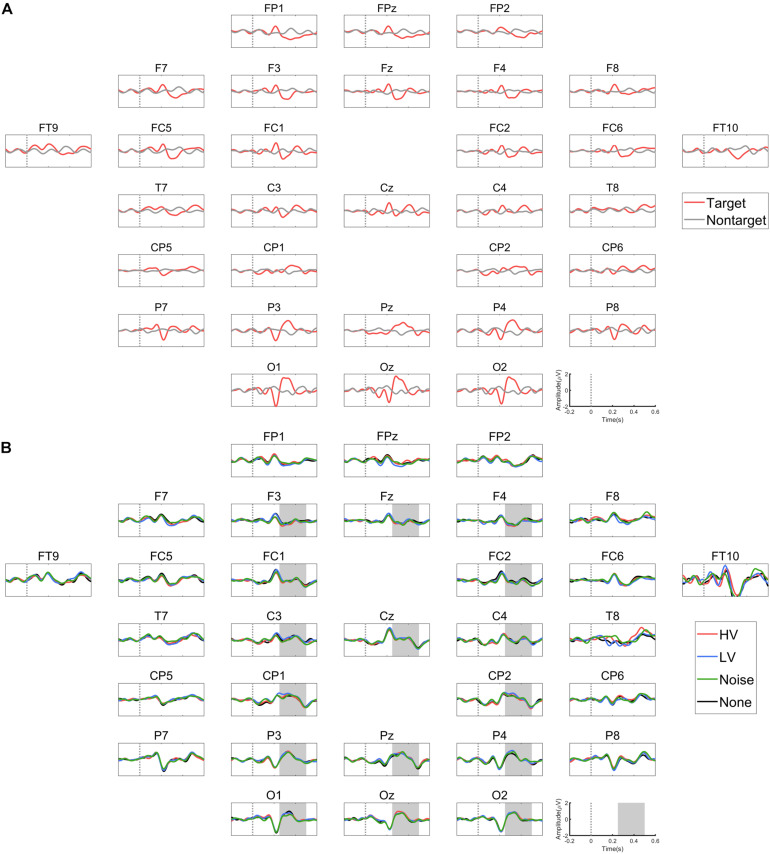
ERP graphs. **(A)** Grand average ERP graphs obtained from the training set. The red line represents ERP of target and black line does that of non-target stimuli. **(B)** Grand average ERP graphs obtained from the test set for each of the four emotional auditory stimulation conditions. The shaded area indicates where the analysis for P300 component was conducted.

We visually inspected the ERPs from the training data to examine whether the P300 component was induced by the target stimulus (Figure 3A). As expected, the P300 component appeared to be present in response to the target but not to the non-target over many channels (e.g., Pz, Oz, and others). Next, we compared the ERPs of different auditory conditions from the test data (Figure 3B, Supplementary Table 3 and Supplementary Table 4). We observed no conspicuous difference between the conditions in the ERP patterns in response to the target stimulus. The rmANOVA was conducted on those channels in which P300 was observed: F3, Fz, F4, FC1, FC2, C3, Cz, C4, CP1, CP2, P3, Pz, P4, O1, Oz, and O2. The rmANOVA revealed no significant difference in the peak ERP amplitude and latency among the conditions except for F3 (Table 1). In order to examine the peak amplitude level at F3, the peak amplitude was compared between the target and non-target stimuli, and a paired *t*-test showed no significant difference for all conditions (HV: *p* = 0.35, LV: *p* = 0.27, Noise: 0.21, None: *p* = 0.26). In addition, we repeated the comparison of the ERP peak amplitude and latency in each group of subjects: the LD and SD groups. For this analysis, we used the Friedman test followed by the Tukey’s-HSD *post hoc* test. The LD group showed a significant difference in the peak amplitude only at channel O1 between the HV and None conditions (HV < None, *p* = 0.02), while it showed no difference in the peak latency. The SD group showed a significant difference between the conditions in neither the peak amplitude nor peak latency (*p* > 0.05).

### Online BCI Performance

**FIGURE 4:**
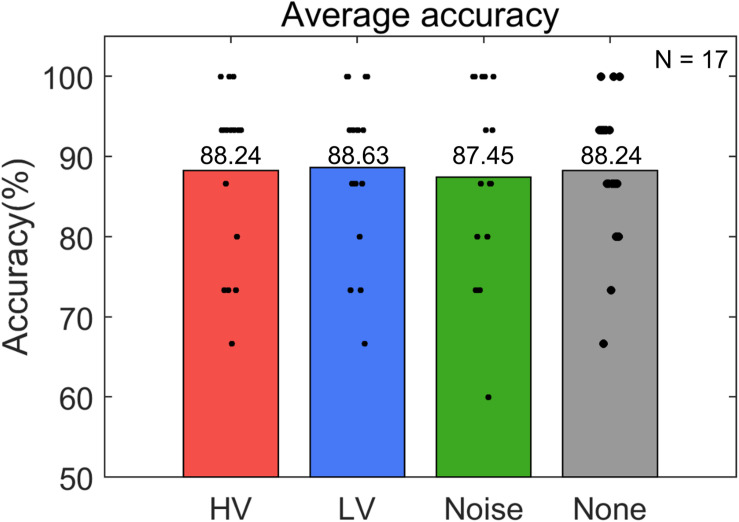
Average accuracy of online P300-based BCI control for each stimulus condition (HV, high valence; LV, low valence; Noise, noise sound; None, no sound). Black dots represent the accuracy of individual subjects in each condition. N indicates the number of samples.

**FIGURE 5:**
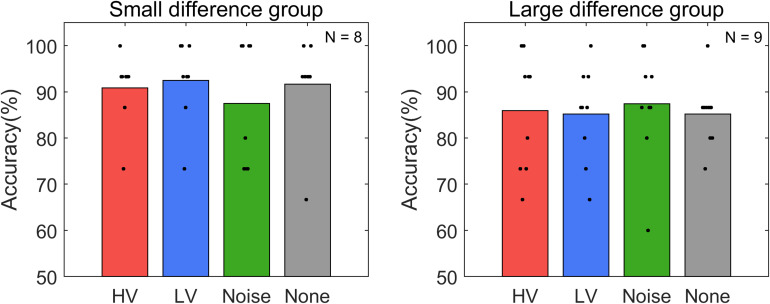
Average accuracy of online P300-based BCI control for each stimulus condition (HV, high valence; LV, low valence; Noise, noise sound; None, no sound) in each subject group: small difference group and large difference group. See the text for the details of the criteria of dividing groups. Black dots represent the accuracy of individual subjects in each condition. N indicates the number of samples in each group.

Subjects operated the P300-based BCI to control the electric light device with an average accuracy of 88.14 ± 7.26% (Figure 4). The maximum and minimum accuracy among subjects was 98.33% and 73.33%, respectively. The rmANOVA showed no significant difference in accuracy between the conditions [*F*(3,48) = 0.086, *p* = 0.98]. The accuracy was also compared in two groups. The average accuracy of the LD group was 85.93 ± 5.15% and that of the SD group was 90.63 ± 8.77%. Wilcoxon rank sum test showed no significant difference between these groups (*p* = 0.118). In addition, The Friedman test did not show any significance between the conditions in either the LD [χ^2^(3, *N* = 8) = 0.89, *p* = 0.828] or SD group [χ^2^ (3, *N* = 9) = 0.49, *p* = 0.922] (Figure 5).

**FIGURE 6:**
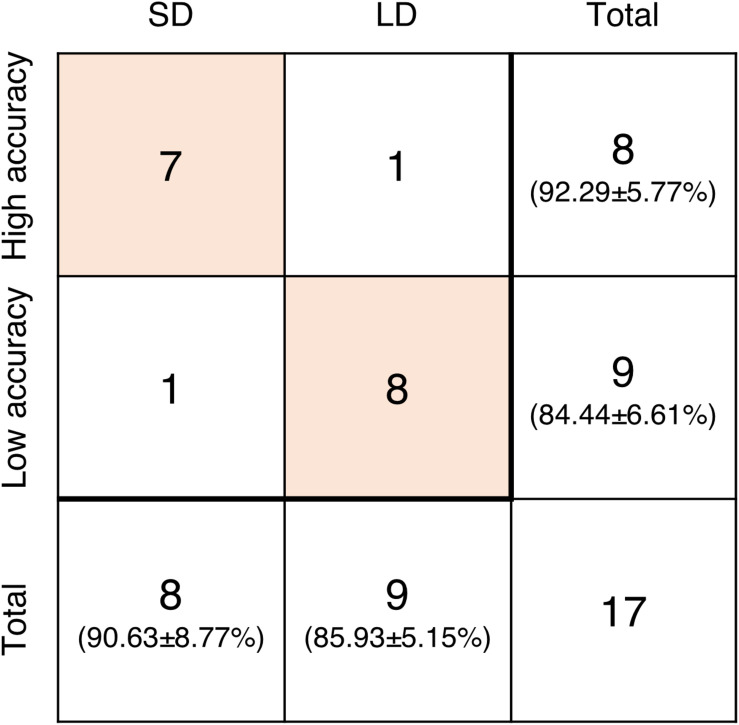
The number of subjects included in groups based on the difference from None condition (SD and LD) and the accuracy in None condition (high accuracy and low accuracy). The values in parentheses are the average accuracy of the corresponding group.

Additionally, subjects were grouped again according to their accuracy in the None condition. Subjects who showed higher accuracy than the average belonged to the high accuracy group and those with lower accuracy than the average belonged to the low accuracy group. The average accuracy of the high accuracy group was 92.29 ± 5.77% and that of the low accuracy group was 84.44 ± 6.61%. Wilcoxon rank sum test showed a significant difference between these groups (*p* = 0.0216). Among eight subjects in the SD group, only one subject was included in the low accuracy group. Similarly, 8 out of 9 subjects in the LD group belonged to the low accuracy group (Figure 6).

### Frontal Alpha Asymmetry

**FIGURE 7:**
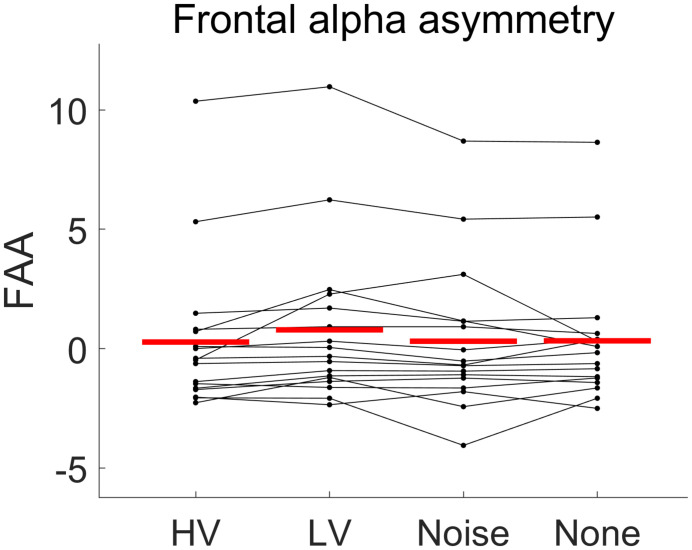
The distributions of frontal alpha asymmetry (FAA) values under each emotional stimulus condition (HV, high valence; LV, low valence; Noise, noise sound; None, no sound). The red line shows the average of FAA value over subjects in each condition. Every line connecting dots represents the FAA variation of each subject across conditions. No significant difference in FAA between the conditions was found [*F*(3,48) = 2.496, *p* = 0.071].

**FIGURE 8:**
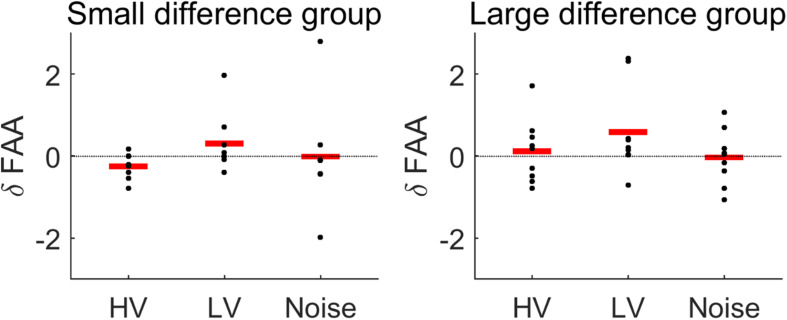
Changes in FAA from the None (no sound) condition to each stimulus condition (HV, high valence; LV, low valence; Noise, noise sound), in each subject group: small difference group and large difference group. See the text for the details of the criteria of dividing groups. Red lines indicate mean changes in FAA. The Wilcoxon signed rank test showed no significant difference between the conditions in each group.

Overall, the rmANOVA revealed no significant difference in FAA between the conditions [*F*(3,48) = 2.496, *p* = 0.071] (Figure 7). In the group-wise analysis, the Wilcoxon signed rank test did not show any significant change of FAA from the None condition to each of the other auditory conditions (HV, LV, and Noise), in either the LD or SD group (*p* > 0.05) (Figure 8 and Table 2).

**TABLE 2:** The statistical test results of changes in frontal alpha asymmetry (FAA) in a given stimulation condition compared to the condition of no sound (Wilcoxon signed rank test).

Subject group	Stimulation condition	Signed-rank statistic	*p*-value
SD	HV	6	0.1094
SD	LV	24	0.4609
SD	Noise	12	0.4609
LD	HV	23	1
LD	LV	38	0.0742
LD	Noise	22	1

*SD, small difference group; LD, large difference group; HV, high valence; LV, low valence.*

## Discussion

In the present study, we investigated the effect of externally induced emotions on the performance of a P300-based BCI. Subjects participating in this study received emotional auditory stimuli designed to induce positive (HV) and negative (LV) emotions while controlling an electric light device through the P300-based BCI. In addition to these emotional stimuli, noise (neutral valence) as well as no sound was presented. We compared the ERP properties, online BCI performance and FAA between the four conditions (HV, LV, Noise, and None). We found no significant difference in the ERP peak amplitude and peak latency over most EEG channels except for F3 (although a difference in the peak amplitude was found at F3, the amplitude level was relatively small and thus hard to extract meaningful results). Also, BCI control accuracy and FAA were not different between the conditions. Subjects controlled the electric light using the BCI fairly well under all conditions (online control accuracy of 88.14% on average). Furthermore, we examined whether the extent to which individuals were influenced by emotional stimuli contributed to individual differences in accuracy. To this end, we divided subjects into two groups based on the difference of accuracy between the emotional and None conditions. We observed no significant difference in BCI control accuracy, ERP peak amplitude and FAA across the conditions within each of the large difference (LD) group and small difference (SD) group. From the results of the present study, there was no clear evidence that emotional stimulations would affect the P300-based BCI performance.

Previous studies have suggested that visual or auditory emotional stimuli can influence P300-based BCIs when the stimuli are used as targets to select (Onishi et al., 2017; Fernandez-Rodríguez et al., 2019; Onishi and Nakagawa, 2019). In these studies, P300-based BCIs included emotional stimuli—such as sounds or images with different valence levels—as task-relevant stimuli, so that the user was attending to those emotional stimuli. This paradigm is different from our study in which emotional stimuli are irrelevant to the task. In our paradigm, the user is attending to emotionless stimuli relevant to the task, while receiving a separate set of task-irrelevant emotional stimuli. Our task paradigm is closer to real-life situations because the user would be exposed to a variety of emotional stimuli from uncontrolled environments when controlling home appliances using BCIs.

In previous studies where background stimuli were present during the use of BCI, the BCI accuracy was not improved, but in most cases decreased (Lian et al., 2017; Voznenko et al., 2018; Cherepanova et al., 2019; Xu et al., 2020). Especially, the visual BCI performance deteriorated when background stimuli, whether auditory or visual, attracted attention. Also, the more mental workload was required, the more the accuracy decreased (Cherepanova et al., 2019; Xu et al., 2020). In addition, the presence of background stimuli without any requirement of attention often showed reduced performance in BCI (Lian et al., 2017; Voznenko et al., 2018). According to Voznenko et al. (2018), music listening while using a BCI influenced each individual differently. Some subjects were negatively affected by music stimuli regardless of the genre of music, whereas others showed the decreased accuracy in specific genre of music. The authors discussed that subjects reported different levels of interference with music depending on their preference, which could cause distraction to the music. Hence, it can be deduced that when background stimuli do not demand mental workload, the effect of them depends on the extent to which people are distracted to them. In our study, the auditory emotional stimuli, which did not demand any attention, did not show significant influence on the BCI performance. It might be because the emotional stimuli did not evoke distraction enough to decrease the BCI performance on average in subjects of this study.

When we narrowed our focus on a subset of subjects showing differences in BCI control accuracy with emotional stimuli, overall BCI control accuracy in the LD group was not different between emotional conditions. This may be because the effect of emotional stimuli on BCI performance could vary over subjects in the LD group. Also, average accuracy in the SD group tended to be higher than in the LD group. SD group, those whose accuracy under emotional conditions did not change from the control condition, tended to be good at operating P300 BCIs. Therefore, good BCI performers might be relatively less influenced by emotional conditions. However, it is still premature to draw any conclusion from this analysis due to the lack of a sufficient number of samples. Therefore, a more in-depth study is necessary to investigate influences of emotional state changes on the use of the BCI specifically for those who are more susceptible to external emotional events.

Even though we asked subjects to rate valence and arousal scores of emotional auditory stimuli independently of BCI control, we additionally computed FAA in each condition to estimate their emotional states during the online BCI control task. FAA has been widely used as a metric to represent emotional valence (Davidson et al., 1979; Harmon-Jones et al., 2010). It was confirmed in our experiment that the valence score of HV stimuli remained positive and that of LV stimuli remained negative before and after the task. We also found no difference between the SD and LD groups in the valence scores for HV and LV stimuli, respectively (*p* > 0.05). In contrast, FAA showed no difference between the HV, LV, Noise, and None conditions. This result of FAA may be associated with no significant difference in ERPs and BCI performance, implying that external emotional stimuli given during BCI control did not induce emotional changes much in the brain. We conjecture that no clear effect of the valence of emotional stimuli on FAA might be due to the fact that subjects were likely to concentrate on selecting targets during the online BCI control session with real-time feedback from the device, which could weaken the effect of auditory emotional stimuli. However, this conjecture would not be made possible if we only look into the survey results as self-reporting on HV or LV stimuli remained positive or negative. In addition, we observed decreases in the absolute valence scores after the BCI control session. This reduced emotional recognition of stimuli intensity may be potentially due to repeated experiences because people tend to habituate to emotional stimuli when those stimuli are repeated and evaluate the repeated emotional stimuli to a smaller degree (Dijksterhuis and Smith, 2002; Leventhal et al., 2007).

In this study, we found no evidence to support a hypothesis that emotional stimuli would influence the performance of P300-based BCIs. However, there are some limitations in this study, which needs further investigations. First, the number of subjects in each group was too small for statistical test results within each group to be considered significant. Future studies with a larger sample size should follow up to confirm our preliminary results. Second, FAA in the HV or LV condition was not increased compared to that in the None condition, which might indicate that the HV or LV auditory stimuli did not successfully evoke positive emotions. If the auditory stimuli had been selected based on FAA combined with self-reports, the effect of emotional stimuli on FAA might be more clearly manifested. This may indicate a need to simultaneously measure FAA during self-reporting on emotional stimuli in future studies. Third, it was plausible that our BCI control task was so intense that subjects’ attention might be mostly attracted to the task and visual processing, leaving little room for the perception of auditory stimuli. To verify this plausibility, we should have a brief session in which we simply provided the prepared set of auditory stimuli to subjects and analyzed ERPs and FAA to confirm that subjects’ emotional state changed. The follow-up studies may need to consider such an addition to experimental design. Lastly, the age range of subjects in our study was below 29 years. Subjects had to attend to the BCI task while the irrelevant auditory stimuli were presenting in the experiment. Since younger people are better at ignoring irrelevant stimuli (McDowd and Filion, 1992), which would worked as one of the strategies to successfully complete the required task, those who are older than subjects in this study may produce different results. To clarify this important inquiry, further studies need to investigate the effect of emotions on P300-based BCIs for elder populations.

Nonetheless, to the best of our knowledge, the present study investigates the effect of emotional stimuli on the online performance of a P300-based BCI for the first time and reveals that there is no significant effect by neither positive nor negative stimuli. We envision that the present study’s results may provide useful information to those who are concerned with potential effects of ambient stimuli when they build a P300-based BCI in practice.

## Data Availability Statement

The raw data supporting the conclusions of this article are available on request to the corresponding author.

## Ethics Statement

The studies involving human participants were reviewed and approved by the Ulsan National Institutes of Science and Technology, Institutional Review Board. The patients/participants provided their written informed consent to participate in this study.

## Author Contributions

MK conducted the experiments, analyzed the data, and wrote the manuscript. DH and JK conducted the experiments, analyzed the data, and wrote the manuscript. YC participated in writing the manuscript. TL designed the experiments and conducted the experiments. S-PK oversaw the study and managed every part of the research. All authors read and approved the final manuscript.

## Conflict of Interest

The authors declare that the research was conducted in the absence of any commercial or financial relationships that could be construed as a potential conflict of interest.
