# Supplementary material for: Effects of Emotional Stimulations on the Online Operation of a P300-Based Brain–Computer Interface
Source: Front Hum Neurosci. 2021 Feb 26;15:612777. doi: 10.3389/fnhum.2021.612777 (PMC7987063; doi:10.3389/fnhum.2021.612777)
Supplement: Supplementary file 1 [file Table_1.DOCX]

Supplementary Material

# Supplementary Tables

**Supplementary Table 1.** Description, means and standard deviations of each dimension (valence, arousal, dominance) of International Affective Digitized Sounds-2 (IADS-2) sound used in the experiment for High valence. All information is from Bradley and Lang, 2007.

| Description | Sound No. | ValMN | ValSD | AroMN | AroSD | DomMN | DomSD |
| --- | --- | --- | --- | --- | --- | --- | --- |
| RockNRoll | 815 | 7.9 | 1.53 | 6.85 | 2.16 | 6.86 | 1.99 |
| Laughing | 226 | 7.78 | 1.37 | 5.42 | 2.13 | 6.32 | 1.82 |
| Bongos | 817 | 7.67 | 1.46 | 7.15 | 2.11 | 6.44 | 1.73 |
| Crowd2 | 311 | 7.65 | 1.58 | 7.12 | 1.83 | 6.09 | 2.18 |
| Baby | 110 | 7.64 | 2.1 | 6.03 | 1.98 | 6.14 | 1.88 |
| Beethoven | 810 | 7.51 | 1.66 | 4.18 | 2.38 | 6.07 | 1.92 |
| Harp | 809 | 7.44 | 1.41 | 3.36 | 1.84 | 6.29 | 1.87 |
| Bach | 811 | 7.4 | 1.63 | 4.95 | 2.46 | 6.14 | 1.87 |
| Baseball | 353 | 7.38 | 1.53 | 6.62 | 1.42 | 6.04 | 1.86 |
| Casino2 | 367 | 7.33 | 1.74 | 6.72 | 2.03 | 6.41 | 1.98 |
| Applause1 | 351 | 7.32 | 1.62 | 5.55 | 2.08 | 6.74 | 1.71 |
| SlotMachine2 | 717 | 7.32 | 1.64 | 6.56 | 2.19 | 6.39 | 2.3 |
| BoyLaugh | 220 | 7.28 | 1.91 | 6 | 1.99 | 5.99 | 1.88 |
| Wedding | 813 | 7.2 | 1.86 | 5.89 | 2.4 | 5.51 | 1.95 |
| SportsCrowd | 352 | 7.17 | 1.97 | 7.07 | 2.12 | 5.77 | 2.08 |
| Robin | 151 | 7.12 | 1.56 | 4.47 | 2.27 | 5.73 | 1.92 |
| Casino1 | 366 | 7.09 | 1.73 | 6.26 | 1.63 | 6.08 | 2.19 |
| Giggling | 230 | 7.05 | 1.44 | 4.84 | 1.86 | 5.77 | 1.55 |
| SlotMachine1 | 716 | 7 | 2.17 | 6.44 | 1.73 | 6.54 | 2.03 |
| Guitar | 816 | 6.98 | 1.9 | 5.23 | 2.08 | 5.84 | 1.88 |
| Party | 365 | 6.97 | 1.9 | 6.32 | 1.9 | 5.73 | 1.76 |
| Seagull | 150 | 6.95 | 1.64 | 4.38 | 2.22 | 5.91 | 1.8 |
| RollerCoaster | 360 | 6.94 | 2.25 | 7.54 | 1.97 | 4.73 | 2.39 |
| FunkMusic | 820 | 6.94 | 1.98 | 5.87 | 1.92 | 5.97 | 1.8 |
| Choir | 812 | 6.9 | 1.69 | 3.43 | 2.56 | 5.69 | 1.9 |
| Kids1 | 112 | 6.84 | 1.72 | 4.46 | 2.13 | 6.07 | 1.68 |
| CorkPour | 726 | 6.82 | 1.6 | 4.51 | 2.08 | 6.36 | 1.71 |
| Crowd4 | 355 | 6.77 | 1.84 | 6.32 | 1.66 | 5.7 | 2 |
| Beer | 721 | 6.71 | 1.75 | 5 | 2.12 | 5.96 | 1.71 |
| Brook | 172 | 6.62 | 1.69 | 3.36 | 2.07 | 6.21 | 1.86 |
| SodaFizz | 725 | 6.61 | 1.8 | 4.55 | 2.17 | 6.3 | 1.95 |
| MaleLaugh | 221 | 6.56 | 1.75 | 5.05 | 1.91 | 5.34 | 1.63 |
| ColonialMusic | 601 | 6.53 | 1.66 | 5.84 | 1.8 | 5.73 | 1.58 |
| Countdown | 415 | 6.46 | 1.67 | 6.55 | 1.56 | 4.8 | 2.25 |
| Carousel | 109 | 6.4 | 2.13 | 5.64 | 1.84 | 5.69 | 1.93 |
| Bugle | 808 | 6.32 | 1.76 | 6.35 | 2.15 | 5.64 | 1.75 |
| BagPipes | 826 | 6.21 | 2.12 | 5.07 | 2.06 | 5.61 | 1.88 |
| Shower | 206 | 6.2 | 1.6 | 4.4 | 1.82 | 5.62 | 1.61 |
| VideoGame | 254 | 6.17 | 1.65 | 5.58 | 1.99 | 6.25 | 2.05 |
| NativeSong | 802 | 6.17 | 1.99 | 5.29 | 1.74 | 5.72 | 1.8 |
| Kids2 | 224 | 6.11 | 1.9 | 5.64 | 1.89 | 5.49 | 1.82 |
| Whistling | 270 | 6.1 | 1.83 | 4.23 | 2.06 | 5.85 | 1.93 |
| HorseRace | 363 | 6.1 | 1.88 | 6.32 | 2 | 5.05 | 1.67 |
| Doorbell | 378 | 6.06 | 2.01 | 6.15 | 2.22 | 5.47 | 1.83 |
| Jet | 400 | 6.02 | 1.49 | 5.38 | 1.87 | 4.86 | 1.86 |
| MusicBox | 111 | 6.01 | 2.19 | 5.65 | 1.91 | 5.42 | 2.02 |
| Polaroid | 375 | 5.99 | 1.6 | 4.48 | 1.74 | 5.67 | 1.95 |
| Thunderstorm | 602 | 5.99 | 2.23 | 3.77 | 1.74 | 4.85 | 2.27 |
| ClapGame | 225 | 5.96 | 1.51 | 4.83 | 1.93 | 5.49 | 1.56 |
| CourtSport | 370 | 5.94 | 1.66 | 4.44 | 1.72 | 5.83 | 1.78 |

ValMN: Valence Mean, ValSD: Valence Standard deviation, AroMN: Arousal Mean, AroSD: Arousal Standard deviation, DomMN: Dominance Mean, DomSD: Dominance Standard deviation

**Supplementary Table 2.** Description, means and standard deviations for each dimension (valence, arousal, dominance) of IADS-2 sound used in the experiment for Low valence. All information is from Bradley and Lang, 2007.

| Description | Sound No. | ValMN | ValSD | AroMN | AroSD | DomMN | DomSD |
| --- | --- | --- | --- | --- | --- | --- | --- |
| ChildAbuse | 278 | 1.57 | 1.43 | 7.27 | 1.6 | 3.49 | 2.48 |
| FemScream3 | 277 | 1.63 | 1.13 | 7.79 | 1.63 | 2.32 | 1.78 |
| Fight1 | 290 | 1.65 | 1.27 | 7.61 | 1.99 | 2.89 | 2.05 |
| Attack1 | 279 | 1.68 | 1.31 | 7.95 | 2.22 | 2.3 | 1.94 |
| Victim | 286 | 1.68 | 1.18 | 7.88 | 1.72 | 2.31 | 2.03 |
| Attack2 | 285 | 1.8 | 1.56 | 7.79 | 2.01 | 2.41 | 2.02 |
| FemScream2 | 276 | 1.93 | 1.63 | 7.77 | 1.5 | 2.69 | 2.02 |
| MaleScream | 292 | 1.99 | 1.41 | 7.28 | 1.74 | 2.82 | 1.78 |
| Attack3 | 284 | 2.01 | 1.48 | 7.05 | 1.65 | 2.99 | 2 |
| BabiesCry | 260 | 2.04 | 1.39 | 6.87 | 2.13 | 3.46 | 2.31 |
| CarWreck | 424 | 2.04 | 1.52 | 7.99 | 1.66 | 2.29 | 1.74 |
| Scream | 275 | 2.05 | 1.62 | 8.16 | 2.15 | 2.55 | 2.01 |
| WomenCrying | 296 | 2.06 | 1.22 | 6.07 | 1.97 | 3.24 | 1.96 |
| Vomit | 255 | 2.08 | 1.78 | 6.59 | 2.08 | 3.23 | 1.98 |
| BikeWreck | 600 | 2.13 | 1.55 | 7.28 | 1.9 | 2.62 | 1.8 |
| Bees | 115 | 2.16 | 1.33 | 7.03 | 1.91 | 2.67 | 1.71 |
| TireSkids | 422 | 2.22 | 1.47 | 7.52 | 1.9 | 2.62 | 1.77 |
| CarHorns | 420 | 2.34 | 1.51 | 7.08 | 2.06 | 2.7 | 1.8 |
| Buzzer | 712 | 2.42 | 1.62 | 7.98 | 1.99 | 2.84 | 2.11 |
| ManWheeze | 244 | 2.44 | 1.34 | 6.31 | 1.85 | 3.16 | 1.97 |
| MaleCough | 241 | 2.46 | 1.53 | 5.87 | 2.06 | 3.52 | 2.07 |
| Siren1 | 711 | 2.61 | 1.59 | 7.39 | 2.02 | 2.93 | 1.82 |
| BusySignal | 703 | 2.65 | 1.59 | 5.68 | 1.89 | 3.26 | 1.92 |
| Creep | 288 | 2.71 | 1.75 | 6.82 | 1.63 | 3.59 | 2.21 |
| PlaneCrash | 501 | 2.74 | 1.76 | 6.93 | 1.91 | 3.12 | 1.96 |
| BabyCry | 261 | 2.75 | 1.68 | 6.51 | 1.96 | 3.91 | 1.97 |
| AlarmClock | 709 | 2.78 | 1.93 | 7.54 | 2.28 | 3.95 | 2.24 |
| FemaleCough | 242 | 2.8 | 1.86 | 5.39 | 1.91 | 3.76 | 1.81 |
| AirRaid | 624 | 2.82 | 1.75 | 7.1 | 2.1 | 3.41 | 2.03 |
| Puppy | 105 | 2.88 | 2.14 | 6.4 | 2.13 | 3.8 | 2.17 |
| DentistDrill | 719 | 2.89 | 1.67 | 6.91 | 2.02 | 2.92 | 2.03 |
| Crash | 732 | 2.89 | 1.68 | 6.98 | 1.75 | 3.32 | 1.88 |
| Fight2 | 282 | 2.92 | 2.34 | 7.2 | 1.63 | 3.92 | 2.31 |
| Sirens | 713 | 2.95 | 1.71 | 6.98 | 1.53 | 3.27 | 1.77 |
| Buzzing | 116 | 3.02 | 1.65 | 6.51 | 2.13 | 4.14 | 2.11 |
| BattleTaps | 611 | 3.02 | 2.06 | 5.34 | 1.75 | 3.67 | 1.99 |
| Fight3 | 283 | 3.05 | 1.72 | 6.2 | 1.6 | 3.85 | 2.05 |
| GunShot | 289 | 3.08 | 1.71 | 6.57 | 1.8 | 3.55 | 2.07 |
| ManSobbing | 293 | 3.08 | 1.92 | 5.74 | 1.69 | 3.94 | 1.82 |
| Siren2 | 714 | 3.1 | 1.67 | 6.94 | 1.85 | 3.56 | 1.73 |
| EngineFailure | 502 | 3.15 | 2.01 | 6.32 | 1.87 | 3.23 | 2.1 |
| GlassBreak | 730 | 3.22 | 1.45 | 6.23 | 1.78 | 4.1 | 1.87 |
| CoupleSobbing | 295 | 3.27 | 2.39 | 5.79 | 1.81 | 3.94 | 2.1 |
| Injury | 423 | 3.31 | 1.79 | 6.23 | 1.6 | 4.22 | 1.89 |
| MayDay | 625 | 3.35 | 2.03 | 6.94 | 1.77 | 3.26 | 2.13 |
| Growl1 | 106 | 3.37 | 1.64 | 6.39 | 1.62 | 3.54 | 1.84 |
| Explosion | 626 | 3.37 | 1.98 | 6.61 | 1.71 | 3.4 | 1.86 |
| Attack3 | 281 | 3.43 | 2.63 | 7.33 | 1.89 | 3.52 | 2.52 |
| MaleSneeze | 250 | 3.54 | 1.57 | 4.94 | 1.9 | 4.08 | 1.67 |
| RattleSnake | 134 | 3.55 | 1.99 | 6.98 | 1.67 | 3.5 | 1.82 |

ValMN: Valence Mean, ValSD: Valence Standard deviation, AroMN: Arousal Mean, AroSD: Arousal Standard deviation, DomMN: Dominance Mean, DomSD: Dominance Standard deviation

# Supplementary Table 3. Average peak amplitude(uV) in the defined time window (250 ~ 500ms) of all channels for each condition (HV, LV, Noise, None)

| Channel | HV | LV | Noise | None |
| --- | --- | --- | --- | --- |
| FP1 | 0.6318 | 0.3492 | 0.4069 | 0.2037 |
| FPz | 0.7301 | 0.5566 | 0.4494 | 0.2463 |
| FP2 | 0.7515 | 0.7053 | 0.329 | 0.4716 |
| F7 | 0.6762 | 0.8476 | 0.7789 | 0.7977 |
| F3 | 0.3443 | 0.7639 | 0.6123 | 0.7897 |
| Fz | 0.374 | 0.6429 | 0.4458 | 0.5194 |
| F4 | 0.5888 | 0.5096 | 0.7308 | 0.6553 |
| F8 | 1.2528 | 0.8228 | 0.591 | 0.7943 |
| FT9 | 0.8459 | 0.9263 | 0.9083 | 0.9182 |
| FC5 | 0.7307 | 0.9291 | 0.9802 | 0.9991 |
| FC1 | 0.5003 | 0.8003 | 0.7746 | 0.6945 |
| FC2 | 0.4699 | 0.6561 | 0.5292 | 0.8123 |
| FC6 | 0.8712 | 0.6816 | 0.7126 | 0.8598 |
| T7 | 0.9204 | 0.9055 | 0.7942 | 0.9765 |
| C3 | 0.8414 | 1.2215 | 1 | 0.9933 |
| Cz | 0.789 | 0.8818 | 0.8247 | 0.8893 |
| C4 | 0.9791 | 0.8725 | 1.0035 | 1.0017 |
| T8 | 1.104 | 0.6869 | 0.158 | 0.547 |
| CP5 | 0.9017 | 1.0468 | 0.9022 | 0.9943 |
| CP1 | 1.1474 | 1.3138 | 1.1755 | 1.1 |
| CP2 | 1.2493 | 1.1492 | 1.0715 | 1.1869 |
| CP6 | 1.3881 | 1.094 | 1.2646 | 1.1856 |
| P7 | 1.0396 | 1.3046 | 0.814 | 0.8571 |
| P3 | 1.4891 | 1.5477 | 1.3935 | 1.5024 |
| Pz | 1.5702 | 1.2409 | 1.4674 | 1.3673 |
| P4 | 1.5204 | 1.5782 | 1.4122 | 1.227 |
| P8 | 1.4351 | 1.3834 | 1.1104 | 1.1139 |
| O1 | 1.3093 | 1.5042 | 1.2562 | 1.6812 |
| Oz | 1.4611 | 1.6056 | 1.3288 | 1.5623 |
| O2 | 1.5039 | 1.6642 | 1.3584 | 1.4525 |

# Supplementary Table 4. Average peak latency(ms) in the defined time window (250 ~ 500ms) of all channels for each condition (HV, LV, Noise, None)

| Channel | HV | LV | Noise | None |
| --- | --- | --- | --- | --- |
| FP1 | 389.2 | 410.1 | 378.5 | 394.1 |
| FPz | 388 | 413.5 | 386.9 | 379.5 |
| FP2 | 415.8 | 397.8 | 355.5 | 359 |
| F7 | 406 | 442.9 | 392.7 | 379.6 |
| F3 | 410.4 | 396.5 | 380.6 | 403.4 |
| Fz | 380 | 377.6 | 385.2 | 402.8 |
| F4 | 407.2 | 408.6 | 409.2 | 412.7 |
| F8 | 409.3 | 391.5 | 376.8 | 374.5 |
| FT9 | 384.2 | 413.5 | 397.5 | 388.4 |
| FC5 | 405.5 | 428.7 | 402.8 | 402.8 |
| FC1 | 397.9 | 383.6 | 395.6 | 386.2 |
| FC2 | 360.7 | 392.8 | 396 | 376 |
| FC6 | 419.2 | 423.2 | 386 | 404.3 |
| T7 | 408.5 | 407.5 | 383.1 | 423.5 |
| C3 | 384.2 | 368.4 | 402 | 384.2 |
| Cz | 359.3 | 372.9 | 366.1 | 372.8 |
| C4 | 373.2 | 365.9 | 382.4 | 376.3 |
| T8 | 425.3 | 418.7 | 344.7 | 377.3 |
| CP5 | 392.5 | 369.2 | 378.8 | 393.1 |
| CP1 | 342.7 | 352.7 | 371.4 | 351.5 |
| CP2 | 347.4 | 358 | 349.9 | 356.5 |
| CP6 | 396.5 | 377.9 | 378.4 | 369.2 |
| P7 | 371.5 | 396.7 | 343.7 | 359.7 |
| P3 | 333.8 | 338.7 | 354.5 | 350.8 |
| Pz | 334.2 | 339.9 | 334.2 | 337.1 |
| P4 | 344.1 | 332.5 | 318.7 | 344.2 |
| P8 | 367.5 | 378.9 | 382.8 | 395.8 |
| O1 | 327.5 | 328.7 | 347.1 | 335.9 |
| Oz | 338.9 | 346 | 337.7 | 352.1 |
| O2 | 323.9 | 351.5 | 337.4 | 358.5 |

**References**

Bradley, M. M. & Lang, P. J. (2007). The International Affective Digitized Sounds (2nd Edition; IADS-2): Affective ratings of sounds and instruction manual. Technical report B-3. University of Florida, Gainesville, FL.
